# Successful treatment of erythema induratum with topical application of antituberculous drugs: A case report

**DOI:** 10.1097/MD.0000000000009010

**Published:** 2017-12-08

**Authors:** Xueling Mei, Junying Zhao

**Affiliations:** Department of Dermatology, Beijing Friendship Hospital, Capital Medical University, Beijing, China.

**Keywords:** cutaneous tuberculosis, erythema induratum, isoniazid, topical application

## Abstract

**Rationale:**

Erythema induratum, a chronic recurrent lobular panniculitis with vasculitis, is strongly associated with Mycobacterium tuberculosis infection. The recommended drugs include isoniazid, rifampicin, and pyrazinamide, which are typically administered in combination (orally or intravenously). Till date, there are no reports about topical application of anti-tuberculous (anti-TB) drugs for treatment of erythema induratum.

**Patient concerns:**

Herein, we present the case of a 73-year-old woman with recurrent ulceration, scarring and obvious pain in her lower legs.

**Diagnoses:**

She was diagnosed of erythema induratum.

**Interventions:**

Topical anti-TB treatment (3.75% isoniazid twice a day) was necessitated by the development of severe gastrointestinal upset and significant reduction in platelets after oral treatment with isoniazid and rifampicin.

**Outcomes:**

The skin lesions showed improvement at one month and remitted mostly at two months. After 6 months, the skin lesions have subsided and no obvious side effects were observed.

**Lessons:**

Our experience may help expand the therapeutic regimens for cutaneous tuberculosis, and provide physicians with alternative options for management of tuberculosis.

## 1 Introduction

Erythema induratum is clinically characterized by tender, erythematous, violaceous nodules, and plaques that are typically seen on the posterior aspect of lower legs, in particular, the calves.^[1]^ Upon diagnosis, an appropriate combination of anti-tuberculous (anti-TB) drugs should be given orally or intravenously over several months.^[2]^ Adverse effects of anti-TB regimens are rather common, such as gastrointestinal symptoms. In this case report, we present the first ever documented case of erythema induratum that was successfully treated with topical administration of anti-TB drugs. The topical administration was necessitated by the development of severe adverse effects to oral anti-TB drugs.

## 2 Case presentation

### 2.1 Past medical history

A 73-year-old woman presented to our hospital with recurrent ulceration and pain in her lower legs. Twenty-seven years ago, the patient had developed a painful coin-sized ulceration on the lateral aspect of her left lower leg. The lesion was diagnosed as erythema induratum by a local physician. The rash gradually subsided with symptomatic treatment (specific medications were unavailable). In 2005, the patient developed painful multiple ulcers on the flexor sides of both legs. In our hospital, the patient was diagnosed as erythema induratum and oral anti-TB treatment with isoniazid and rifampin was prescribed for a month. The symptoms considerably improved, and the treatment was subsequently halted. Six months ago, she again developed nodules on her lower legs, which were associated with ulceration, scarring, and obvious pain, accompanied by 5 kg weight loss. Besides erythema induratum, the patient has a 20-year-long history of vitiligo and a 10-year-long history of hypertension.

### 2.2 Dermatological examination

**Figure 1:**
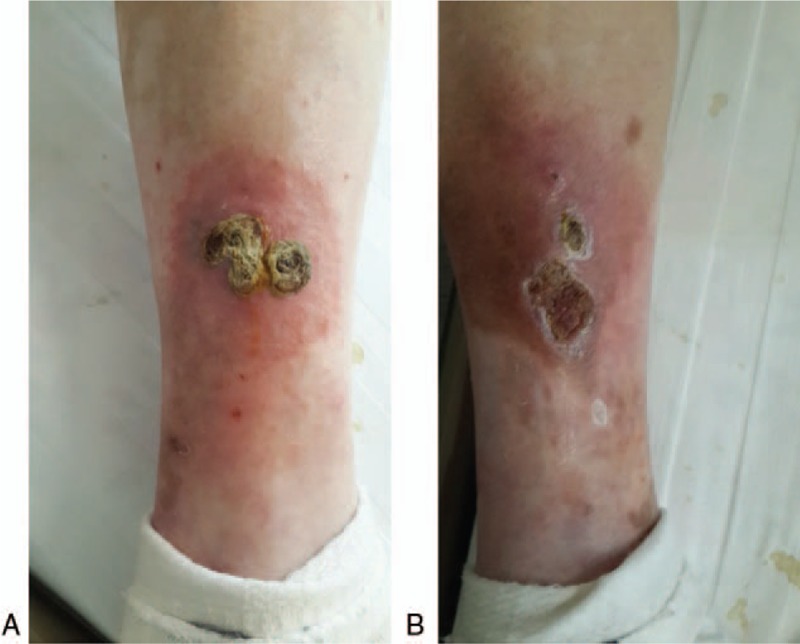
Gross photographs of the lesions affecting both lower legs, (A) two adjacent coin-sized deep-seated nodules are seen on the flexor aspect of the right lower leg; (B) two adjacent dark red nodules are seen on the posterolateral side of the left lower leg.

Two adjacent coin-sized deep-seated nodules were observed on the flexor aspect of the right lower leg; the surface of the nodules was covered with thick yellow-brown crusts (Fig. 1A). Two adjacent dark red nodules, 1.0–2.0 × 2.0 cm, were seen on the posterolateral aspect of the left lower leg; the nodules were tender, had slightly elevated edges, and were covered with thick yellowish-brown crusts (Fig. 1B).

### 2.3 Laboratory examination

Routine blood and urine tests were unremarkable. Biochemical C21 test showed no obvious abnormalities. Chest computed tomography (CT) examination showed nodular calcification in the left lung apex. The tuberculin purified protein derivative test (PPD) was strongly positive (induration of 25 mm with swelling). Acid-fast bacilli were not found on repeated sputum examination.

### 2.4 Histopathological examination

**Figure 2:**
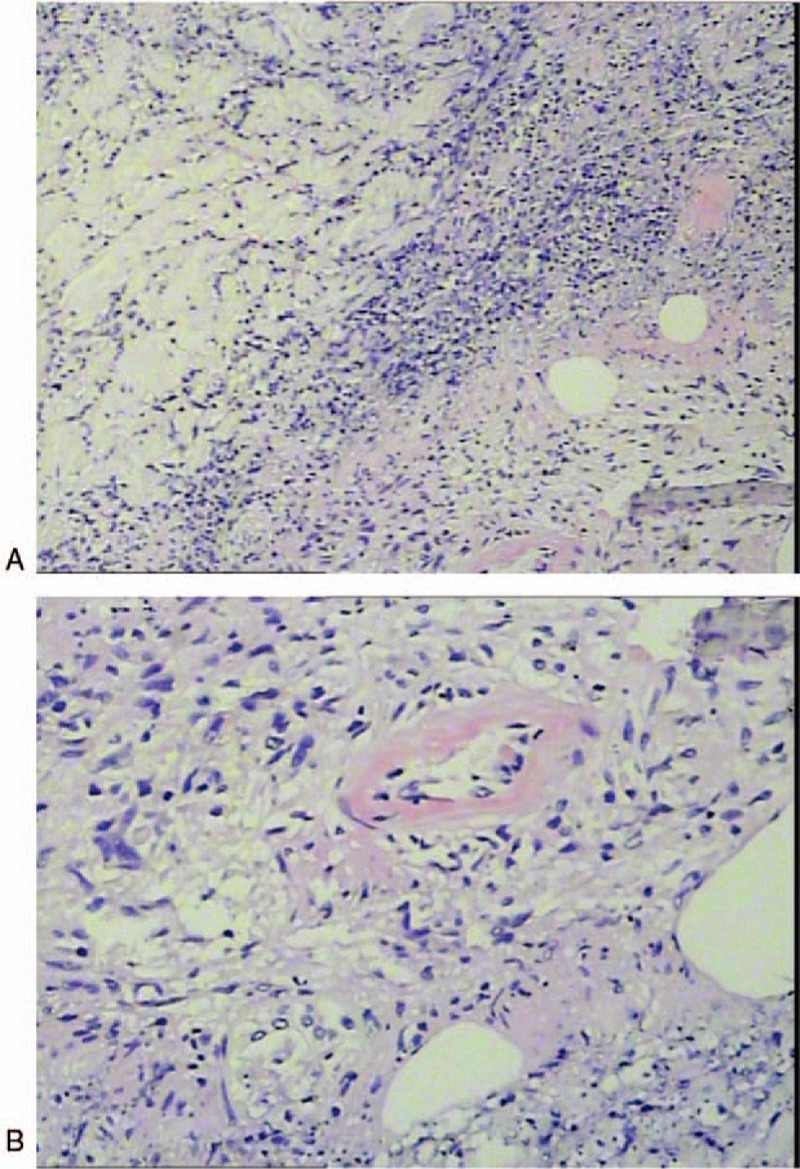
Histopathological examination of biopsy specimen. (A) 4 × 10 HE. (B) 10 × 10 HE.

Biopsy of the lesions showed septal panniculitis with infiltration of neutrophils, vasculitis, inflammatory cell infiltration and necrosis in the lobular adipose tissue, vascular wall thickening in fat space, fibrinoid degeneration, vascular obliteration, and perivascular infiltration of lymphocytes, histiocytes, and a large number of neutrophils. There were no tuberculoid granulomas (Fig. 2).

### 2.5 Diagnosis

Erythema induratum.

### 2.6 Treatment

After definitive diagnosis of erythema induratum with TB, the patient was treated with oral anti-TB therapy, including isoniazid (300 mg qd) and rifampicin (450 mg qd). The patient complained of nausea and vomiting 14 days after initiation of treatment and the blood platelet count was reduced to 20 × 10^9^/L. On suspicion of thrombocytopenia induced by anti-tuberculosis drugs, the anti-TB treatment was immediately stopped. The blood platelet count returned to normal level (98 × 10^9^/L) 1 week after discontinuation of treatment.

**Figure 3:**
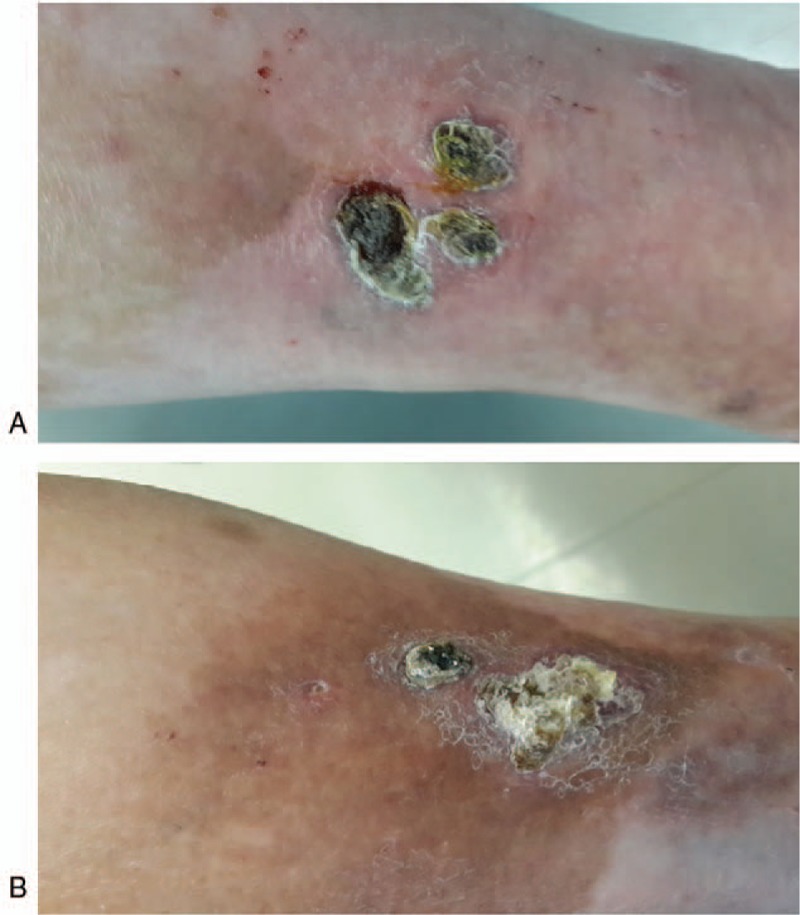
Skin lesions were improved after topical treatment for 1 month. (A) Three dark broad bean size nodules on the flexor side of the left lower leg. (B) Two red nodules on the posterolateral side of the left lower leg.

**Figure 4:**
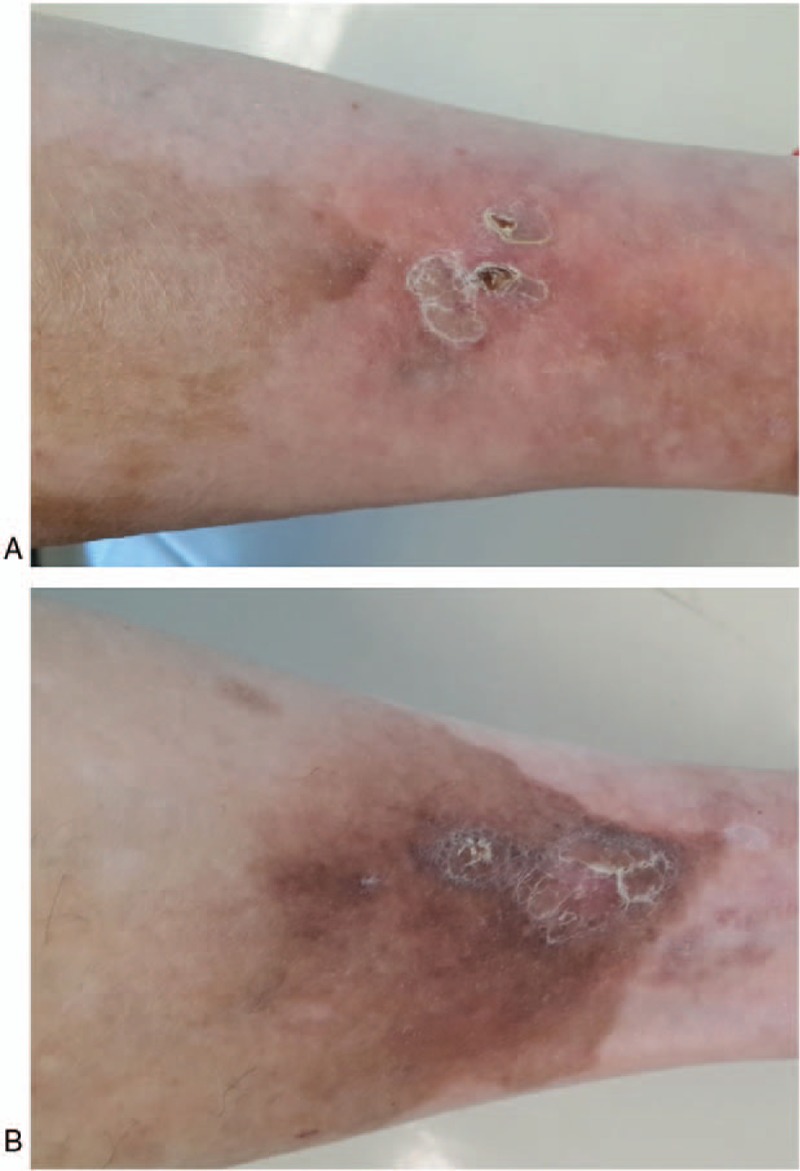
Marked improvement in the skin lesions at 6 months after topical treatment. (A) Flake pigmentation spots on the flexor side of the right lower leg; (B) reddish spots and pigmentation on the lateral side of the left lower leg.

After obtaining informed consent of the patient, we crushed and mixed isoniazid (15 tablets, 1500 mg) with 40 g zinc oxide ointment to prepare a topical anti-TB cream with 3.75% isoniazid. The cream was applied on the skin lesions twice a day (in the morning and the evening). One month later, skin lesions showed improvement (Fig. 3). Two months later, most lesions had remitted. To date, the patient has been topically treated for 6 months, and the skin lesions have subsided (Fig. 4). We plan to continue the treatment for another 3 months, and follow-up the patient thereafter. During the course of treatment, no side-effects such as nausea and vomiting were observed. The platelet count remained within normal range, and liver and kidney functions remained normal.

## 3 Discussion

Multidrug anti-TB therapy is suggested for cases associated with *M tuberculosis*.^[3,4]^ The current recommended treatment regimens for patients with culture-positive TB consist of either a 6-month-long 4-drug course (2 months of RIPE [rifampin, isoniazid, pyrazinamide, and ethambutol], followed by 4 months of isoniazid and rifampin) or a 9-month-long 3-drug course (2 months of rifampin, isoniazid, and pyrazinamide, followed by 7 months of rifampin and isoniazid).^[5]^ Schneider and Jordaan^[1]^ have confirmed the effectiveness of these regimens even in the absence of proven TB.

For treatment of erythema induratum, anti-TB drugs are usually administered orally or intravenously. However, certain side effects of anti-TB drugs, especially gastrointestinal and hematological toxicity, are commonly observed. The patient reported herein developed nausea, vomiting, and significant reduction in platelet count after treatment with oral isoniazid and rifampicin; therefore, oral anti-TB treatment was discontinued. We mixed isoniazid with zinc oxide and applied it on the affected areas as an experimental treatment; interestingly, a curative effect was achieved and no significant side-effects were observed.

To conclude, we report a case of erythema induratum, which was successfully treated with topical application of anti-TB drugs. To our knowledge, this is the first report on the topical treatment of erythema induratum with anti-TB drugs, which may supplement the therapeutic regimens for such diseases.
